# The sensitivity to replacement and displacement of the eyes region in early adolescence, young and later adulthood

**DOI:** 10.3389/fpsyg.2015.01164

**Published:** 2015-08-11

**Authors:** Bozana Meinhardt-Injac, Malte Persike, Margarete Imhof, Günter Meinhardt

**Affiliations:** Department of Psychology, Johannes Gutenberg University MainzMainz, Germany

**Keywords:** development, aging, face perception, configural processing, inversion effect, response bias

## Abstract

Recent evidence suggests a rather gradual developmental trajectory for processing vertical relational face information, lasting well into late adolescence (de Heering and Schlitz, 2008). Results from another recent study (Tanaka et al., 2014) indicate that children and young adolescents use a smaller spatial integration field for faces than do adults, which particularly affects assessment of long-range vertical relations. Here we studied sensitivity to replacement of eyes and eyebrows (F), variation of inter-eye distance (H), and eye height (V) in young adolescents (11–12 years), young (21–25 years), and middle-age adults (51–62 years). In order to provide a baseline for potential age effects the sensitivity to all three types of face manipulations was calibrated to equal levels for the young adults group. Both the young adolescents and the middle-age adults showed substantially lower sensitivity compared to young adults, but only the young adolescents had selective impairment for V relational changes. Their inversion effects were at similar levels for all types of face manipulations, while in both adult groups the inversion effects for V were considerably stronger than for H or F changes. These results suggest that young adolescents use a limited spatial integration field for faces, and have not reached a mature state in processing vertical configural cues. The H–V asymmetry of inversion effects found for both adult groups indicates that adults integrate across the whole face when they view upright stimuli. However, the notably lower sensitivity of middle-age adults for all types of face manipulations, which was accompanied by a strong general “same” bias, suggests early age-related decline in attending cues for facial difference.

## 1. Introduction

The ability to perceive and to recognize faces continuously develops during life-span, steeply rising in infancy and childhood, reaching highest performance in adulthood, and declining with age (Germine et al., 2011). Face perception is regarded as a special domain of ability, because there is no other object category with a comparable degree of part integration (Maurer et al., 2002). However, the high degree of interdependence among face parts is bound to the upright orientation. Turning faces upside down, or even rotating them, disrupts part integration, and sets up a part-wise access to facial features (Thompson, 1980; Tanaka and Farah, 1993; Tanaka and Sengco, 1997; Rossion and Boremanse, 2008). Further, face inversion dramatically affects the ability to judge spatial relations among facial features (Diamond and Carey, 1986; Barton et al., 2001). Some years ago Goffaux and Rossion found an asymmetry in the inversion effects for horizontal relational and vertical relational manipulations of the eyes region (Goffaux and Rossion, 2007). Manipulating vertical relations (changing eye height by moving the eyes and eyebrows region, V) produced large inversion effects, while manipulating horizontal relations (changing eye distance, H) produced small effects of inversion, which were in the same order of magnitude as featural changes (replacement of eyes, F). Sekunova and Barton (2008) contributed and validated a plausible explanation for this asymmetry. Judging eye distance is possible with just a pair of eyes, and without the embedding facial context. Hence, a local analysis of the highly salient eyes region is sufficient to judge eye distance. Eye height, in contrast, cannot be judged with a pair of eyes alone, but needs embedding context. Judging eye height gains precision if long-range spatial relations to multiple face regions (forehead, mouth, nose) are simultaneously taken into account. If inversion narrows the attentional window toward mostly the highly salient eyes region, local relational analysis should be maintained, but distal relational analysis should be affected. Hence, an asymmetry of H and V inversion effects should result. The authors obtained empirical support for their conjecture by testing the effects of moving eye but not eyebrow height. Doing so adds a valid local eye-height cue (eye–eyebrow distance), which can be handled in a small attentional window centered around the eyes. Indeed, this manipulation yielded small inversion effects for eye height (V), in the same order of magnitude as found for eye distance (H).

There is further evidence that the asymmetry in the inversion effects for H and V relational manipulations of the eyes region reflects that local and global configural information are analyzed in parallel by distinct routines when upright faces are viewed. Meinhardt-Injac et al. (2011) found that also the timing prerequisites for H and V inversion effects are quite different. Inversion effects for V appear already at brief timings starting with the first 50 ms, while H inversion effects emerge later, needing exposure durations of at at least 200 ms. Studying the influence of spatial scale Goffaux (2008) found that H manipulations were detected best with high-pass filtered images above 32 cycles per face width (cpfw), while sensitivity to V manipulations were best in bandpass filtered images maintaining the optimal spectrum for faces in the range of 8–32 cpfw. These results indicate that mechanisms sensitive to H manipulations analyse on smaller spatial scales and have sustained temporal characteristics, while mechanisms sensitive to V manipulations analyse on larger spatial scales are instantly responding. Studying the interaction among mouth and eyes region with a context congruency paradigm Goffaux (2009) found that the contextual interaction among these distal face regions was much stronger in the low spatial frequency range below 8 cpfw than in the high spatial frequency range beyond 32 cpfw. Inversion canceled the contextual interaction among both face regions. These results substantiate that the long-range interaction among face parts is critically bound to the upright orientation.

The parallel integration of diagnostic cues from short-range and long-range relations is a remarkable capability of mature adult face vision (Smith et al., 2005). In a recent developmental study by de Heering and Schlitz (2012) the developmental trajectory of the sensitivity for vertical relational image manipulations of the eyes and mouth region was studied in upright faces. The authors observed a gradual, steady increase in the ability to detect changes in eye height (eyes and eyebrows) from 6 to 16 years of age, while detection of different mouth–nose distances remained at low performance levels, improving at a marginal rate across age. These results suggest that judging vertical relations undergoes protracted development, and still does not reach adult levels during adolescence. Tanaka et al. (2014) studied the sensitivity to size changes of mouth and eyes, as well as to relational changes in both regions [eye distance (H) and nose-mouth distance (V)], in the age range of 7–12 years, and compared to adult performance. They found that accuracy for children and young adolescents was remarkably worse than for adults for both featural and relational manipulations. Sensitivity for manipulations of the eyes region did not improve from 7 to 12 years, while sensitivity to manipulations of the mouth region smoothly increased, but at low absolute levels for mouth-nose distance (V). Both studies indicate that efficient use of vertical relational cues in less salient regions of the face is not at mature levels for adolescent observers. The studies add to the findings which support protracted development of encoding relational face properties, which is a key characteristic of the mature “expert” face system (Maurer et al., 2002; Mondloch et al., 2004). Currently, there is no study which addressed whether the typical asymmetry in the inversion effects for H and V is found at younger ages. Because this asymmetry is highly diagnostic for short-range and long-range cue usage in faces (s. above), a study which fills this gap is requisite.

While the H–V asymmetry of inversion effects has yet not been addressed in childhood or adolescence, it has been studied at mature ages (Chaby et al., 2011). The authors used the same relational image manipulations as Goffaux and Rossion (2007), and obtained a large inversion effect for V and a small one for H with older subject in the age range of 60–80 years (mean age 69.9 years). However, the authors found that variation of eye distance (H) for upright stimuli was detected by the elderly with an accuracy near chance. Therefore, the inversion effect for H was limited by a floor effect in the baseline, disabling a valid comparison of H and V inversion effects at mature ages.

In the present study the effects of featural and relational image manipulations of the eyes region were investigated with upright and inverted faces for young adolescents (11–13 years), young adults (21–25 years), and middle-age observers (51–62 years). The age groups were selected such that no ceiling or floor effects could be expected. Further, the image manipulations for all three change types were calibrated to yield equal performance for upright faces in the young adults group. This guaranteed an equal baseline for judging inversion effects across change types, as well as a standard for age-related effects. Doing so, we aimed at revealing relevant clues to the developmental state of young adolescents in handling short- and long-range spatial relations, as well as age-related decline in these abilities.

## 2. Materials and methods

### 2.1. Experimental outline

The sensitivity to featural, horizontal relational, and vertical relational image manipulations of the eyes region was measured, having subjects perform a same/different forced choice task on a sequence of two face images with equal duration. Same and different pairs were equally likely, and stimulus pairs with F, H, and V manipulations were presented in randomly interleaved trials. Further, the stimulus orientation was upright or upside-down, in random alternation. Same pairs were also constructed from two manipulated stimuli in order to preclude that the deviation from the anthropometric face normal could be used as a cue to the difference of face pairs. Catch trials with manipulation of the mouth region were included to have the observers not artificially narrow their attentional focus to just the eyes region. Accuracy and response bias were analyzed using the signal detection paradigm.

### 2.2. Participants

This study was conducted with participants from three age groups: young adolescents (11–12 years), young adults (21–25 years), and middle-age adults (51–62 years). No subject had prior psychophysical experience. Young adolescents (*N* = 20, 10 female, mean age = 11.7 years) were six-grade students of a German grammar school. In the young adults group there were 25 participants (13 female, mean age = 23.3 years). All were students at the Johannes Gutenberg University Mainz, but were not students of psychology. The group of middle-age adults consisted of 20 participants (15 female, mean age = 55.5 years). All participants had normal or corrected-to-normal vision. Furthermore, there were no known psychological conditions present in the participants. Prior to the study, all potential participants, and in case of the young adolescents, also their parents, were informed about the general study aims, the experimental testing approach, and the kind of judgements which were required from them. From all participants (or their parents in case of the young adolescents) written consent was received for participation. All participants participated on a voluntary basis and were not paid for their participation.

### 2.3. Stimuli and calibration for equal salience of F, H, and V manipulations

**Figure 1:**
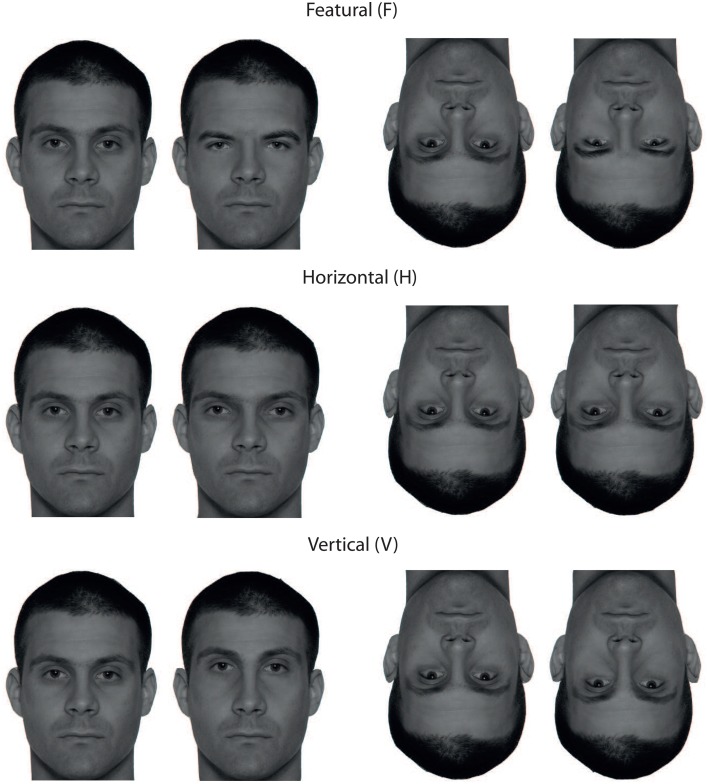
**Illustration of featural (F), horizontal relational (H), and vertical relational (V) differences of a sample face in upright (left) and inverted (right) presentation**.

Photographs of 16 swiss male adults (mean age = 24.6 years, age span = 20−29 years), taken under controlled lighting conditions in a professional photo studio, were used for stimulus construction. The photographs were carefully selected from a larger database with the constraint that manipulation of the eyes and eyebrow region should be possible without evoking the impression of strong facial “oddity” in single stimulus instances. The photographs were converted to 300 × 400 pixel gray-scale images and equalized in contrast. Image manipulations were done using Adobe Photoshop. For featural manipulations the eyes/eyebrows region of a face was replaced with the corresponding region of another face, assuring that no additional position or size cues were introduced by the replacement. In pilot experimentation prior to the main experiment with five student aids exchange pairs were found that yielded about 90% correct same/different judgements. Various values were probed for the horizontal and vertical shift of the eye/eyesbrows region. Finally, moving the eyes and eyebrows 20 pixels apart (H) and 14 pixels upward (V) was found to yield the same proportion of correct judgements as the featural manipulations. These values were used in the main experiment. Stimulus examples of the three change types are given in Figure 1,^1^.

### 2.4. Design

The experiment had a 3 (Change Type) × 2 (Orientation) × 3 (Age) factorial design. The same/different matching task comprised 16 same and 16 different trials in each condition. Each of the 16 face instances was presented with each of the 3 change types once as a same and once as a different pair. We added 24 catch trials where the mouth was replaced, or moved horizontally, or vertically. Combined with trial-by-trial acoustical feedback catch trials were used to preclude that only the eyes region was attended. Each subject completed 216 trials, which lasted about 20 min.

### 2.5. Apparatus

The experiment was executed with Inquisit 2.0 runtime units. Patterns were displayed on NEC Spectra View 2090 TFT displays in 1280 × 1024 resolution at a refresh rate of 60 Hz. Screen background was the same light gray as the face image background. The room was darkened so that the ambient illumination approximately matched the illumination on the screen. Viewing was binocularly at a distance of 70 cm. Stimulus patterns subtended 12 × 15 cm of the screen. Subjects used a distance marker but no chin rest. They gave responses via the left and the right button of the computer mouse. The assignment of answers (same/different) to the left or right mouse button was counterbalanced across participants. Trial-by-trial acoustical feedback about correctness was given via light headphones. Non-annoying sounds were used: a “tack”-tone indicated a correct response, and a “tacktack”-tone signaled an error.

### 2.6. Procedure

The temporal order of events in a trial sequence was: fixation mark (300 ms)-blank (100 ms)-first stimulus frame (633 ms)-mask (350 ms)-blank (200 ms)-second stimulus frame (633 ms)-mask (350 ms)-blank frame until response. Different trials were formed by pairing an original face with a manipulated face, with the assignment of a stimulus to the first or the second place in the trial sequence chosen at random. Same trials were formed by pairing two original faces or two manipulated faces, each alternative with equal likelihood^2^. Masking of the stimulus frames was done with spatial noise patterns with a grain resolution of 3 pixels. The presentation positions of each of the two face images were shifted by 20 pixels away from the center in random direction in order to preclude focusing on the same image parts. Pairs with manipulations according to either change type were presented randomly interleaved. Faces were presented upright or upside down, in random alternation.

The setting for the duration of the stimulus presentations (633 ms) was found in pilot experimentation prior to the main experiment. Five student aids, two young adolescents and two middle-age adults were tested with various exposure durations, ranging from 300 to 1200 ms. The students reached saturating performance already for timings of beyond 400 ms. Accuracy of the two middle-age subjects and the two young adolescents did not further improve for values beyond 633 ms. We therefore decided to select this value for the exposure duration of the face stimuli in the main experiment.

The young adolescents were introduced to the experiment in greater detail. An outline of this study was presented to all grade six pupils at a German grammar school. The investigator explained the general study outline and presented examples of the stimuli on an overhead projector. She clarified in detail with different face image examples why stimulus pairs were the same or different. Each participant received an additional individual explanation prior to the experiment. Here, four faces on a piece of paper with the same face template but in all four conformations (original, F, H, and V) were shown. To reassure that the face manipulations were understood the participants were asked to point out why the faces were different. Subsequently they were invited to start with first test trial on the computer, using the computer mouse for the responses as in the later experiment. After a short introduction by the experimenter the subjects initiated each probe trial on their own. After that, each participant completed 36 probe trials in order to ensure that the instruction was understood and could be put into practice. For young and middle-age adults the same individual explanation procedure was used to ensure that subjects unfamiliar with psychophysical tasks were equally well instructed. Specifically, all participants were informed that two instances of the same basic face would appear in a sequence, either identical or slightly differing in the inner part of the face. Participants were also informed that occasional changes in the mouth region could occur, which should not be overlooked. Participants were told to give any answer if they were uncertain about the right alternative, but to try to be as correct as possible.

### 2.7. Performance measures

Accuracy was measured in terms of the proportion of correct judgments for each response alternative and then transformed to *d*′ using standard formulae, i.e., *d*′ = *z*(*Hit*)−*z*(*FA*) for the sensitivity measure and *c* = −1∕2(*z*(*Hit*)+*z*(*FA*)) for the response criterion on the standard axis, scaled such that 0 referred to no response preference, negative values indicated a “same” bias and positive values a “different” bias. Since the “same” response category is commonly defined as the target category in the recent face perception literature (e.g., Richler et al., 2011) we complied with this standard. Accordingly, hit-rate (Hit) was defined as the rate of correctly identifying “same” trials and correct rejection rate (CR) was defined as the rate of correctly identifying “different trials.” False alarm rate (FA) and the rate of misses (Miss) were defined as being the complementary rates to CR and Hit, respectively.

### 2.8. Data analysis

Both the *d*′ measure and the response criterion *c* were analyzed with ANOVA, having age group (Age) as grouping factors and change type (Change Type) and orientation (Orientation) as repeated measurement factors. To reveal effects of Change Type in the sensitivity measure separate ANOVAs per age group were run, since Change Type was calibrated for performance equivalence in the younger adults group, and this might underestimate the true variance of the Change Type factor in the main effect and its interactions with Orientation and Age.

## 3. Results

### 3.1. Sensitivity measure

**Figure 2:**
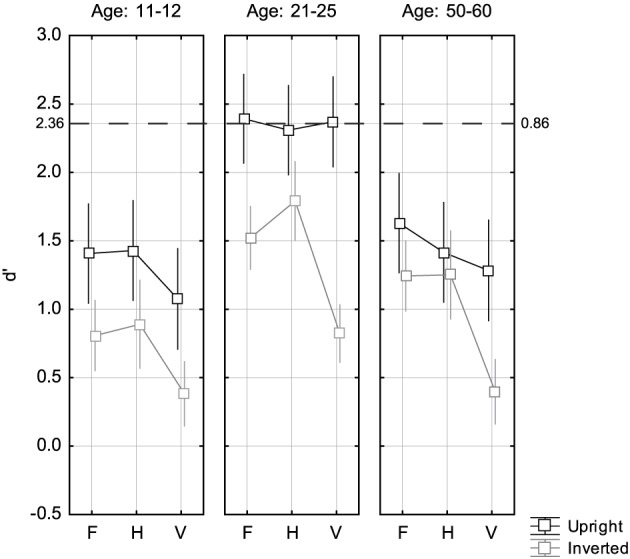
**Mean accuracy (***d***′) for the three change types F, H, and V in the three age groups**. Black squares indicate data for upright presentation, and gray squares refer to inverted presentation. Error bars indicate 95% confidence limits of the means. The dashed line indicates the reference level of performance from the young adults group, which was calibrated for equivalence across change types F, H, and V. On the right axis, this level is indicated as proportion correct, and as *d*′ on the left axis.

Figure 2 shows the average *d*′ scores for the three age groups and the three change types. The data for the young adults reflect equal performance for upright faces with F, H, and V manipulations at a level of 86% correct, which corresponded to a *d*′ score of 2.36. This value was slightly below the target calibration value of 90% correct, which was reached by a subgroup of experienced observers of the same age in pilot experimentation. Overall ANOVA confirmed that there were substantial main effects of Age, Change Type, and Orientation (see Table 1). As indicated by significant interactions, the effect of Orientation was modulated by Age and by Change Type, while the interaction among all three factors was marginally significant. The interaction of Change Type and Age was not significant. The assumption of normality was checked for the ANOVA data by analyzing normality of the within-cell residuals with the q–q plot correlation technique (Filliben, 1975). This test showed fairly good agreement of *d*′ residuals with the assumption of normality (see Appendix).

**Table 1:** **Overall ANOVA results for the same/different matching accuracy for faces (***d***′ measure) in three age groups**.

**Source of variation**	**SS**	***df***	**${\hat{\sigma }}^{2}$**	**F**	**p**	**${\hat{\eta }}^{2}$**
Age (A)	56.80	2	28.40	19.82	< 0.001	0.390
Error	88.83	62	1.43			
Change Type (B)	17.55	2	8.78	23.59	< 0.001	0.276
Change Type × Age	0.81	4	0.20	0.55	0.701	0.017
Error	46.13	124	0.37			
Orientation (C)	45.82	1	45.82	102.01	< 0.001	0.622
Orientation × Age	4.61	2	2.30	5.13	0.009	0.142
Error	27.85	62	0.45			
Change Type × Orientation	6.73	2	3.37	13.48	< 0.001	0.179
A × B × C	2.24	4	0.56	2.24	0.069	0.067
Error	30.97	124	0.25			

*The table shows source of variation, sum of squares (SS), degrees of freedom (df), variance estimate (${\hat{\sigma }}^{2}$), F- ratio (F), significance level (p), and partial eta-squared ($\eta_{p}^{2}$)*.

*Post-hoc* testing with Fisher LSD tests showed that younger adults outperformed young adolescents and middle-age adults both with upright and inverted stimuli (all *p* < 0.001). With either orientation, performance was not significantly different among young adolescents and middle-age adults [*p* = 0.435 (upright), *p* = 0.129 (inverted)].

To further explore the marginally significant interaction among all three factors separate ANOVAs were run for each age group. The results are shown in the Tables 2–4. In the young adolescents group there were strong main effects of Change Type and Orientation, but no interaction among both factors. LSD *post-hoc* tests showed that performance in V was worse than in H (*p* < 0.001) and F (*p* < 0.002), while performance in H and F was at equal levels (*p* = 0.635). For both young and middle-age adults there were strong main effects of Change Type and Orientation and a strong interaction among both factors. LSD *post-hoc* tests indicated worse performance in V compared to H (young adults: *p* < 0.001; middle-age adults: *p* < 0.005) and F (young adults: *p* < 0.004; middle-age adults: *p* < 0.001), and no different performance in H and F (young adults: *p* < 0.43; middle-age adults: *p* = 0.533). Note that, for young adults, these differences just reflected the change type effects for inverted stimuli.

**Table 2:** **ANOVA results for the same/different matching accuracy for faces (***d***′ measure) in the young adolescents group**.

**Source of variation**	**SS**	***df***	**${\hat{\sigma }}^{2}$**	**F**	**p**	**${\hat{\eta }}^{2}$**
Change Type	4.42	2	2.21	9.24	0.001	0.327
Error	9.10	38	0.24			
Orientation	11.18	1	11.18	32.95	< 0.001	0.634
Error	6.45	19	0.34			
Change Type × Orientation	0.12	2	0.06	0.21	0.810	0.011
Error	10.89	38	0.29			

*Conventions as in Table 1*.

**Table 3:** **ANOVA results for the same/different matching accuracy for faces (***d***′ measure) in the young adults group**.

**Source of variation**	**SS**	***df***	**${\hat{\sigma }}^{2}$**	**F**	**p**	**${\hat{\eta }}^{2}$**
Change Type	5.75	2	2.87	8.29	0.001	0.257
Error	16.64	48	0.35			
Orientation	35.91	1	35.91	85.36	< 0.001	0.781
Error	10.10	24	0.42			
Change Type × Orientation	6.84	2	3.42	15.34	< 0.001	0.390
Error	10.71	48	0.22			

*Conventions as in Table 1*.

**Table 4:** **ANOVA results for the same/different matching accuracy for faces (***d***′ measure) in the middle-age adults group**.

**Source of variation**	**SS**	***df***	**${\hat{\sigma }}^{2}$**	**F**	**p**	**${\hat{\eta }}^{2}$**
Change Type	8.13	2	4.07	7.57	0.002	0.285
Error	20.40	38	0.54			
Orientation	6.88	1	6.88	11.56	0.003	0.378
Error	11.30	19	0.59			
Change Type × Orientation	2.74	2	1.37	5.55	0.008	0.226
Error	9.38	38	0.25			

*Conventions as in Table 1*.

### 3.2. Inversion effects

The overall ANOVA indicated that inversion effects were strongly modulated by age. The specific age dependency of the inversion effects is best reflected in the separate ANOVAs for each age group (see Tables 2–4). For both young and middle-age adults the inversion effect was strongly modulated by Change Type (see Tables 3, 4) while, for young adolescents, the inversion effect was independent of Change Type (see Table 2).

**Figure 3:**
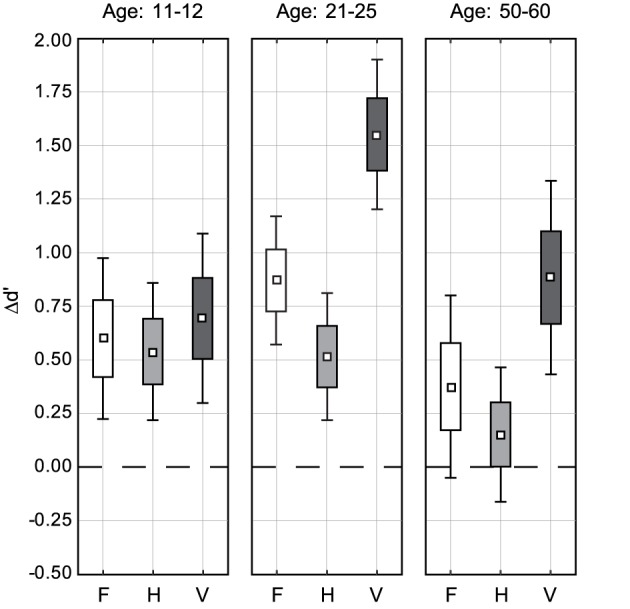
**Face inversion effects for the three change types F, H, and V in the three age groups, represented by Box–Whisker plots**. The inner box represents the mean IE, the outer box standard error and the Whiskers indicate 95% confidence limits of the mean IE. Note that a IE is significant if 0 (dashed black horizontal line) is outside the confidence interval.

To better illustrate the effects of face inversion we calculated IEs at the level of individual data, and showed the results as Box–Whisker plots (Figure 3). The difference data were also fed into ANOVA in order to allow for *post-hoc* comparisons across conditions and age groups^3^. These analyses substantiated that in both adult groups there was practically the same results pattern of IEs, while young adolescents showed different IE results.

As it was expected from the non significant Change Type × Orientation interaction for young adolescents, LSD *post-hoc* tests showed that inversion effects were at about the same levels for F, H, and V (F vs. H: *p* = 0.787; F vs. V: *p* = 0.674; H vs. V: *p* = 0.489). In contrast, for both adults groups the inversion effect was the strongest for vertically manipulated faces (young adults: V vs. F and V vs. H both *p* < 0.001; middle-age adults: V vs. F and V vs. H both *p* < 0.03). The IEs for F tended to be larger than the IEs for H, but with just marginal significance for young adults (*p* = 0.079) and failing statistical significance (*p* = 0.323) for middle-age adults.

LSD *post-hoc* comparisons across age showed that the IE of young adults in V was significantly larger than any other IE (*p* < 0.005 for the test against the IE in V for middle-age adults and *p* < 0.001 for any other pairwise comparison). For F and H young adolescents and young adults reached IEs at comparable levels (all *p*>0.25). Evaluating confidence intervals (see Figure 3) showed that, for middle-age adults, the IEs for F and H were moderate, failing significance for H [*F*_(1, 19)_ = 1.22, *p* = 0.284] and reaching just marginal significance for F [*F*_(1, 19)_ = 3.66, *p* = 0.071]. However, *post-hoc* comparison against the corresponding IEs for young adolescents gave non-significant results (F: *p* = 0.397; H: *p* = 0.138). Comparing against the IEs of young adults revealed a significantly larger IE of young adults in F (*p* < 0.04) but not in H (*p* = 0.140). This might reflect the limited testing power of *post-hoc* testing, particularly when a difference measure is used DeGutis et al. (2013).

### 3.3. Response bias

**Figure 4:**
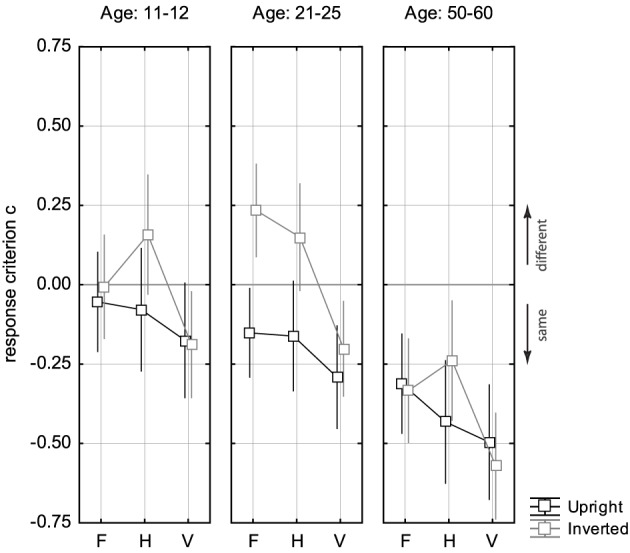
**Response criterion ***c*** for the three change types F, H, and V in the three age groups**. Black squares indicate data for upright presentation, and gray squares refer to inverted presentation. The expected value 0 (bias-free response) is accentuated by a solid gray line. Error bars indicate 95% confidence limits of the means.

Analysing the response criterion *c* as the indicator of response bias (see Figure 4) revealed significant differences between young adolescents, young and middle-age adults [Age: *F*_(2, 62)_ = 11.28, *p* < 0.001]. Middle-age adults showed a strong general “same”-bias, while young adults and young adolescents did not [young adolescents: *F*_(1, 19)_ = 0.97, *p* = 0.336; young adults: *F*_(1, 24)_ = 1.82, *p* = 0.190; middle-age adults: *F*_(1, 19)_ = 46.46, *p* < 0.001]. Also stimulus orientation modulated the subjects' response preferences [*F*_(1, 62)_ = 10.14, *p* < 0.003], since inverted faces more often elicited “different” responses than did upright faces. This response pattern was most pronounced in young adults, while, in the other two age groups, this tendency was negligible [Age × Orientation: *F*_(2, 62)_ = 3.16, *p* < 0.05; LSD *post-hoc*: *p* = 0.216 (young adolescents), *p* < 0.001 (young adults), *p* = 0.659 (middle-age adults)]. In all three age groups the preference for “same” responses increased in the order F, H, V [Change Type: *F*_(2, 124)_ = 18.05, *p* < 0.001; Change Type × Age: *F*_(4, 124)_ = 0.57, *p* = 0.687]. Analysis of the catch trials showed rather low percentage correct in each of the three age groups: 58.4% (young adolescents), 69.2% (young adults), 62.7% (middle-age adults). This indicates that the mouth region was not in the active window of spatial attention, albeit the catch trials should alert the observers also to attending the lower face part.

## 4. Discussion

We revisited the inversion effect for F, H, and V face image manipulations in the eyes region for young adults, and compared with young adolescents and middle-age observers. The sensitivity for detecting changes according to the three change types was calibrated to an equal level (*d*′ = 2.36) in the young adults group. Both the young adolescents and the middle-age adults showed an about 1 *d*′ unit lower sensitivity. For young adolescents the decline was strongest for V relational manipulations. Inversion effects for young adults showed the typical H–V asymmetry, with strongest IEs for V and moderate ones for H, while IEs for F were at intermediate levels. This exactly replicated previous results (Meinhardt-Injac et al., 2011). For middle-age adults nearly the same IE results were found, but with a constantly smaller IE magnitude. Inversion effects for young adolescents, however, did not show the H–V asymmetry, and were at equal levels for F, H, and V. In the following, the findings are discussed for each age group. Finally, we give an outlook to current constraints for inversion effect measurement across age.

### 4.1. Young adolescents show lowered sensitivity to vertical relational changes in the eyes region

The generally lowered sensitivity level of more than 1 *d*′ unit indicates that young adolescents are still far away from adult levels in their ability to judge featural and relational face image manipulations of the eyes region. Our results correspond to findings of Tanaka et al. (2014), who also found generally lowered sensitivity to featural and relational changes at younger ages up to 12 years. In our study sensitivity to changes in eye height (V) was particularly lowered, while sensitivity to eye distance (H) was larger, at the same level as for replacement of eyes and eyebrows (F). Mondloch et al. (2002) mixed manipulations of eye height and eye distance (“configural”) and compared to replacement of eyes and mouth (“featural”). They found that sensitivity to featural manipulations improved faster with age than sensitivity to configural manipulations. Disentangling H and V relational changes shows that eye distance and featural changes in the eyes region are detected equally well by young adolescents at 10–12 years of age (this study; Tanaka et al., 2014). Both de Heering and Schlitz (2008) and Tanaka et al. (2014) found that vertical relational manipulations in the mouth region were detected with relatively poor sensitivity in the age range of 7–12 years. Featural and relational manipulations of the eyes region were found to be detected much better. However, in both studies the locus of change (mouth region, eyes region) and the type of relational change (H, V) were not orthogonally varied, which makes it difficult to judge whether protracted development concerns V type relational changes, compared to H relational changes, or the mouth region compared to the eyes region. Because Tanaka et al. (2014) observed that the sensitivity to featural changes in the mouth region was as high as the sensitivity to H relational changes in the eyes region, one might conclude that sensitivity to V relational changes develops more slowly (de Heering and Schlitz, 2008).

### 4.2. No asymmetry of the inversion effect for H and V relational changes in the eyes region for young adolescents

Adults show a pronounced IE asymmetry for H and V relational manipulations of the eyes region (Goffaux and Rossion, 2007; Sekunova and Barton, 2008; Meinhardt-Injac et al., 2011). We found that young adolescents do not show this typical asymmetry, but exhibit equal inversion effects for F, H, and V. According to Sekunova and Barton (2008) eye distance can be judged without further reference to distal contextual cues, while judging eye height necessarily relies on reference to other facial features and should therefore improve by integrating relational cues across the whole face. If inversion narrows the spatial window of cue integration to a region centered around the eyes, judgement of eye height should suffer more than judgement of eye distance. Also the sensitivity to changes in non-salient, distal face regions should strongly decline. The “spatial narrowing” hypothesis of inversion is supported by findings which show that the IE for non-salient face regions is generally large, but declines substantially if the observers are cued to the region of interest, or a blocking design is used, or observers are given enough time to scan the face stimulus part by part (Barton et al., 2001; Sekunova and Barton, 2008). In line with this interpretation of the inversion effect, the observation of same IEs for all change types indicates that the spatial window of cue integration of young adolescents is confined to a limited region centered around the eyes. Since judging eye height critically depends on cue integration from multiple face regions, the sensitivity of young adolescents to vertical relational changes is disproportionally lowered compared to adults, who integrate cues from the whole face in upright face vision. Inversion further shrinks the window of cue integration, but this should concern detection of F, H, V changes to equal degrees if cue integration for upright stimuli is already confined to the eyes region.

Hence, both findings, the disproportionately lowered sensitivity to V changes in the eyes region and the lack of the asymmetry in the inversion effects for H and V relational changes support the conjecture of Tanaka et al. (2014) that the window of facial cue integration is centered to a confined region around the eyes during childhood, but widens during the course of development, ending in the ability of young adults to simultaneously integrate local and distal cues across the whole face.

### 4.3. The effects of featural changes

The question whether there are distinct mechanisms tuned to “features” and “configurations” has raised serious quarrels in face processing literature (Riesenhuber et al., 2004; Rossion, 2008; Riesenhuber and Wolff, 2009). In a recent review of the magnitude of the inversion effect including 22 studies McKone and Yovel (2009) reported that inversion effects for featural changes were small only when non-shape properties, such as color or brightness, were changed. For shape changes inversion effects were found to be in the same order of magnitude as for manipulations of feature spacing. Most critical for the size of the IE was involvement of facial context. These results confirm to us that a sound distinction of featural and configural processing is impossible (see also Discussion in Meinhardt-Injac et al., 2011). Shape changes do necessarily alter the relational description of a face stimulus—but what authors generally mean by “featural” changes are structural changes of features (scaling, replacement) and *not* changes of color, contrast or glare.

For both adult groups we found stronger inversion effects for replacement of the eyes region (F) than for manipulating eye distance (H). This indicates that replacement of eyes and eyebrows alters the relational description of face stimuli stronger than moving eyes apart. Note that a change of eyes and eyebrows is usually accompanied by a change of personal identity, while moving eyes apart is not. The “featural” change in Figure 1 is readily perceived for the upright face pair, but not for the inverted (upper row). The difference in eye distance is still salient for the upside-down pair (mid row), indicating the relative contextual independence of eye distance. The F change in the upright pair is salient because one sees two different persons, and not just two different pairs of eyes. The stronger inversion effect for F compared to H for adults results from holistic integration across the face, which suffers from shrinking the spatial focus due to inversion. Young adolescents do not show this effect—but exhibit same inversion effects for all three change types. This, again, corroborates that their spatial integration window is confined to the eyes region, while the area of integration spans the whole face in adults. Therefore, we conclude that the effective area of cue integration is a simple concept with potentially much higher explanatory power than the “featural-configural” dichotomy, which is not validated in terms of inversion effect.

### 4.4. Sensitivity to F, H, and V manipulations in middle-age adults

In a recent cross-sectional study Germine et al. (2011) found evidence for a late peak of face memory performance. Using the Cambridge Face Memory Test they found a performance peak at about 30 years, and continuous decline afterwards. Interestingly, face inversion effects showed an increase up to middle adult ages. In this study, middle-age adults performed at approximately the same level as young adolescents when comparing upright stimuli with featural and relational manipulations of the eyes region. This means that there is a remarkable age-related decline in this ability in the age range of 50–60 years. Chaby et al. (2011) compared the sensitivity to H and V manipulations of the eyes region among young adults and older participants (mean age 69.9 years). For young adults their results exactly correspond to our measurements, with a mean accuracy of slightly below 90% in upright presentation, a very large IE for V changes and a moderate one for H changes. For older adults, they obtained about 75% correct in upright presentations for V, which again corresponds to our results, but chance performance for H. In our study middle-age adults were able to handle H changes with at least equal accuracy than V changes. A further difference to our results is that V changes were detected at chance level for inverted stimuli in both age groups in the Chaby study, while, here, performance was well above chance in all experimental conditions. Chaby et al. (2011) claimed that their finding of a large IE for V, which was comparable to the IE of young adults, indicated that configural processing along the important vertical face axis encompassing eyes and mouth region is maintained at mature ages.

Besides the puzzling inability to judge H relations, the conclusion that vertical relations are preserved at mature ages is not fully supported by the measurements of the Chaby study, since chance level performance with inverted stimuli in both age groups implies that the true size of the inversion effect is not revealed. It can therefore not be excluded that the true inversion effect of young adults is larger. Because V relational changes were realized by manipulating both eye height and mouth height, shrinking the window of facial cue integration by inversion can account for the strong IE in both age groups. The confined window would no longer encompass the mouth region, and half of the spacing difference would stay unnoticed in upside-down stimuli.

The decline in sensitivity of about 1 *d*′ unit for upright stimuli observed in this study speaks against the claim that a full and flexible use of long- and short-range relational cues is maintained at mature adult ages. Indeed, we found the typical asymmetry in the inversion effects for H and V changes, but all IEs were smaller compared to young adults. As for the young adults, the H–V asymmetry of the inversion effect suggests that also middle-age adults integrate relational cues across a large face area for upright stimuli and use a confined integration window for upside-down faces. However, middle-age subjects performed notably worse than young adults with *upright* stimuli, while the performance difference with inverted stimuli was considerably smaller (see Figure 2). This suggests that there is an age-related difference in the efficiency of using diagnostic cues, which are in principle available, since the cue integration window is wide. These results correspond to findings of Daniel and Bentin (2012), who found that adults at mature ages show decline in applying configural information in gender categorization based on internal features, a task that heavily relies on an appropriately using local-configural cues. Studying the interaction of external and internal features with a congruency paradigm Meinhardt-Injac et al. (2014b) found the same degree of contextual interaction for young adults and elderly, indicating holistic integration across the whole face for upright stimuli in both age groups. Older adults, however, suffered from a loss of precision when handling internal features. Roudaia et al. (2008) studied contour integration performance and obtained results which suggest that aging is accompanied by a loss in elementary local grouping mechanisms. While there is increasing evidence that the general holistic nature of face perception is maintained at mature ages (Konar et al., 2013; Meinhardt-Injac et al., 2014b), recent findings suggest that adapting viewing strategies aided by feedback, coping with increased attentional demand and flexible handling of diagnostic cues are affected by aging (Meinhardt-Injac et al., 2014a).

### 4.5. Response bias effects across age

Analysis of response bias revealed an interesting age effect. Middle-age adults were strongly biased toward “same” responses, while young adults and adolescents had no global response preference. A global bias toward “same” responses was also reported for older subjects in the age range of 65–78 years for the composite face task (Meinhardt-Injac et al., 2014a). This indicates that the most frequent error of older adults in face comparisons is overlooking the difference. This tendency might result from the failure to attend the relevant diagnostic features, and a loss of detail precision (see above). Also the type of image manipulation modulated response bias. Vertical relational judgements were accompanied by the strongest “same” bias across all ages. For V changes the response criterion *c* was consistently lower than for F and H changes for all age groups, and it was also below the expected value 0, which indicates that there was an absolute bias for “same” responses, and not only a relative tendency compared to H and F (see Figure 4). Hence, for V changes there was an age-independent tendency to overlook the difference in feature spacings. The general “same” bias for V changes is a further hint that the cues that mediate detection of the difference are not all in the active window of spatial attention. We added catch trials with changes in the mouth region in order to preclude that subjects attended only the eyes region. The poor accuracy in the catch trials is a hint that subjects nonetheless mostly concentrated on the eyes region. This indicates that relational cues from the distal mouth region surely entered with minor weight in the same/different judgement of two faces^4^.

### 4.6. Studying sensitivity to relational changes across age

The results of this study suggest that the distinction of H and V configural changes is much more relevant for hypothetical developmental trajectories than the “featural” and “relational” dichotomy. Compared to horizontal relations, the ability to judge vertical relations seemingly suffers from a developmental delay, which is yet not balanced in early adolescence. However, comparing sensitivity to H and V relations is confounded with the effective size of the spatial cue integration window. At the time, it is unclear whether the poorer performance of young adolescents in judging V relations is due to a smaller area of facial cue integration, or the processing route for vertical configural information (Goffaux et al., 2009) has not yet fully matured, or, likely, both reasons apply. The results for young adolescents obtained here suggest both a smaller cue integration field and a specific developmental delay for processing V relations. The results for middle-age subjects suggest a wide cue integration field, but a general sensitivity decline for configural cues. Further research should address ways to disentangle the two hypothetical sources of less efficient facial cue integration by applying techniques which allow to selectively estimate the area of facial cue integration. The bubbles-technique (Gosselin and Schyns, 2001) would offer a possible way to go.

## Ethics statement

This study was carried out in accordance with the Declaration of Helsinki. The experimental procedures were approved by the local ethics committee at the Johannes Gutenberg University Mainz. All subjects participated voluntarily and were informed that they were free to stop the experiment at any time without negative consequences. Written informed consent was obtained from all participants, in case of children, consent was also obtained from the parents. The data were analyzed anonymously.

## Author contributions

All authors contributed equally to the conceptualization of the study. BI and MI set up the basic design. MP conducted the experiments and data preparation. GM contributed data analysis and interpretation. All authors were involved in writing, preparation of the manuscript and final approval. All authors agree to be accountable for all aspects of the work in ensuring that questions related to the accuracy or integrity of any part of the work are investigated and resolved appropriately.

## Funding

This study was supported by the university research fund of Johannes Gutenberg University Mainz. Funding was granted to BM for project “Visual perception across the life-span.”

### Conflict of interest statement

The authors declare that the research was conducted in the absence of any commercial or financial relationships that could be construed as a potential conflict of interest.

## Figures and Tables

**Figure A1:**
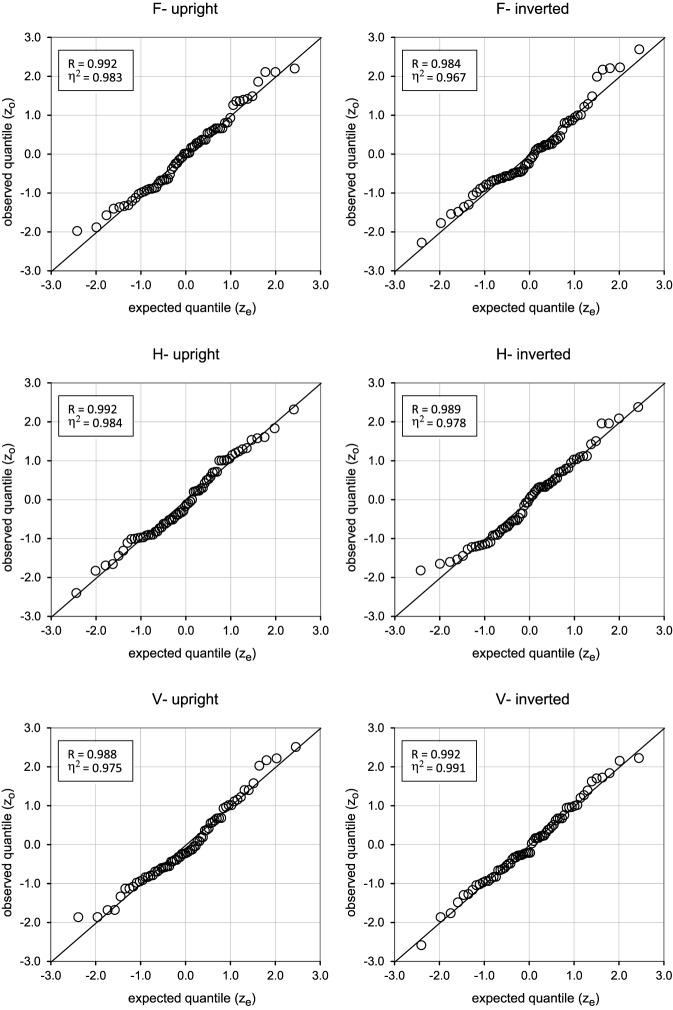
**Quantile–Quantile scatterplots (q–q plots) of the standardized within-cell residuals for the six experimental conditions**. The straight line is the angle bisector, *z*_*o*_ = *z*_*e*_. The figure legends show the Pearson correlation *R* of *z*_*o*_ and *z*_*e*_, and the ratio of explained variance for the identity of observed and expected *z*-scores, η^2^.

## Footnotes

^1^We decided to use just male face models, because the attractiveness of the female faces was particularly affected by changing metric proportions. There is evidence that the range of acceptable facial proportions is narrower in attractive faces (Grammer and Thornhill, 1994; Green et al., 2008). Various studies have substantiated an own-age bias in face recognition (see Rhodes and Anastasi, 2012, for an overview). However, there are only few studies studies on the cross-age effect for face perception tasks. In line with an expertise account of face perception de Heering and Rossion (2014) observed that young adults had a (slightly) larger composite face effect for faces of their own age, as compared to child faces. The effect was not found with preschool teachers. Wiese et al. (2013) studied a potential own-age bias with the composite face task in young (mean age 22.4 years) and older (mean age 67.8 years) adults. They found that both age groups had a small but significant performance advantage with young adult faces. The behavioral effect corresponded to slightly larger N170 amplitudes. Hence, no evidence for a stimulus age × participant age interaction was found. In view of these results we conclude that there is currently no indication for a strong own-age bias in the ability to apply holistic and/or configural viewing strategies to faces when purely perceptual tasks without long-term memory load are used.

^2^We paired also two manipulated faces in same trials to preclude that perception of facial “oddity” (i.e., deviation from the anthropometric average) could be used as a cue to the difference of face pairs. This was used as a means to having the observers compare just the perceptual impressions of the faces, without referring to what is experienced as “normal.” We do not use quantitative descriptions of normative facial anthropometric descriptions (Farkas, 1994), since at least the V manipulations would require to refer to a variety of index measures which change in close correspondence to a variation of eye height (forehead length, nose length, eyes-to-nose distance, etc.). Potentially relevant contextual cues for judging eye height are discussed in this article.

^3^Note that the main effects and interactions of the IE difference data are already included in the overall ANOVA.

^4^Note that the “spatial window of cue integration” is not necessarily identical with the attended face region. If the eyes region is attended, and the mouth region is not, cues from the mouth are also perceived, but with less precision. Context congruency paradigms exploit that there is a perceptual interaction among attended and non-attended face parts (Goffaux, 2009; Meinhardt-Injac et al., 2010).

## Appendix

### Testing ANOVA cell-residuals for normality

In order to check the assumption of normality for the ANOVA data the within-cell residuals were standardized and the agreement of observed *z*-scores (*z*_*o*_) and the *z*-scores expected from the standard normal distribution (*z*_*e*_), was assessed via the q–q plot correlation technique (Filliben, 1975). Figure A1 shows the q–q plots for the six experimental conditions, including the Pearson correlation coefficient, *R*, and the proportion of explained variance for the parameter-free function *z*_*o*_ = *z*_*e*_, denoted as η^2^. Comparing the correlation coefficients to the critical correlation value for *N* = 75 observations, *R*_*crit*, 5%_ = 0.984 (see Johnson and Wichern, 2003, p. 182) shows that there was no violation of normality in any of the six experimental conditions.
